# 

**Published:** 1913-02

**Authors:** 


### CONTENTS.

ORIGINAL COMMUNICATIONS:—

Inaugural Address. By J. Edgar Paullin, M. D., Atlanta, Ga. .... 551

"What Can We Do to Be Saved?" By Samuel A. Visanska, Ph. G., M. D., Atlanta, Ga. .... 554

The Fascination of Opthalmology. By Cecil Stockard, M. D., Atlanta, Ga. .... 561

The Right Kidney—Disquieting Factor in the Diagnosis of Acute Intra-Abdominal Conditions. By W. L. Peple, Richmond, Va. .... 566

EDITORIAL .... 576

SELECTIONS AND ABSTRACTS .... 578

NEWS AND NOTES .... 589

BOOK REVIEWS .... 593

